# Supplementary material for: The posterior parietal cortex contributes to visuomotor processing for saccades in blindsight macaques
Source: Commun Biol. 2021 Mar 4;4:278. doi: 10.1038/s42003-021-01804-z (PMC7933420; doi:10.1038/s42003-021-01804-z)
Supplement: Supplementary file 2 — Supplementary Information [file 42003_2021_1804_MOESM2_ESM.pdf]

Supplementary Information

The posterior parietal cortex contributes to visuomotor processing for saccades in blindsight macaques

Rikako Kato, Takuya Hayashi, Kayo Onoe, Masatoshi Yoshida, Hideo Tsukada, Hirotaka Onoe, Tadashi Isa, Takuro Ikeda

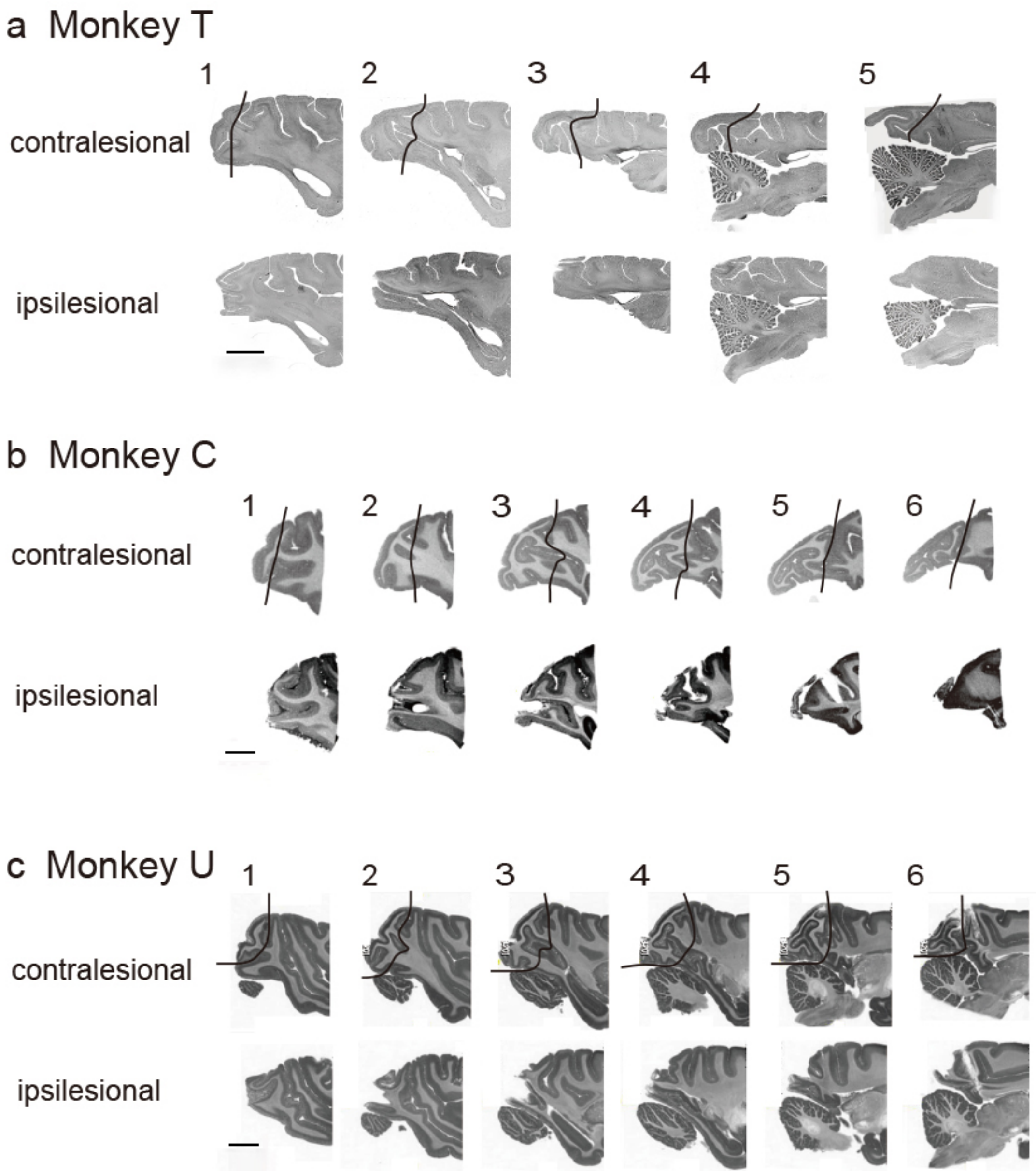

Supplementary Figure 1. Extent of lesions.

a; Nissl-stained parasagittal sections of posterior part of the brain of monkey T; contralesional side in the upper panels and ipsilesional side in the lower panels. The most lateral plane on the left and the most medial plane on the right. As judged from comparison between the contralesional and ipsilesional sides, presumed extent of lesion at individual laterality on the ipsilesional side is indicated on the corresponding contralesional planes as left to the curved black lines in each panel. **b** and **c**; MRI images of the parasagittal planes of the formalin-fixed postmortem brains of monkey C and U,

respectively. The same arrangement as **a**. **a2**, **b3** and **c3** in this figure correspond to the planes of **Fig. 1d**, **f** and **h** in the main text, respectively. All the scale bars indicate 10 mm.

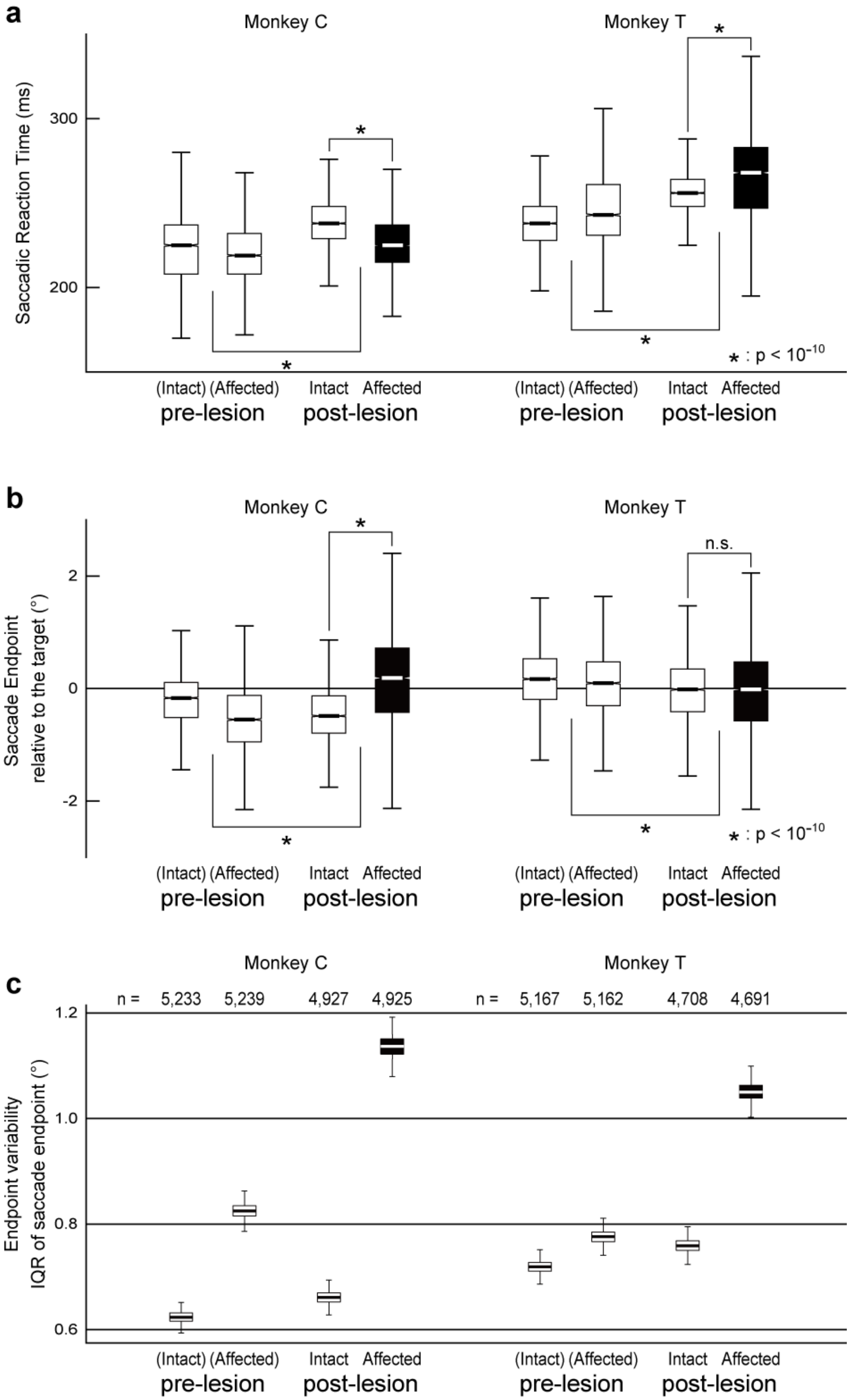
a
Monkey C
Monkey T
Saccadic Reaction Time (ms)
300
200
*
*
*
*
* : p < 10^-10
(Intact) (Affected)
Intact Affected
pre-lesion
post-lesion
b
Saccade Endpoint
relative to the target (°)
2
0
-2
n.s.
c
n = 5,233 5,239 4,927 4,925
n = 5,167 5,162 4,708 4,691
Endpoint variability
IQR of saccade endpoint (°)
1.2
1.0
0.8
0.6

Supplementary Figure 2. Behavioral results: round saccade task.

a. Box plots showing the median and 1st and 3rd quartiles of saccadic reaction times in each condition. Whiskers represent 1.5 interquartile range. Saccades to the affected visual fields are represented by black boxes. **b.** Saccade accuracy (saccade endpoint relative to target location, horizontal direction). **c.** Saccade endpoint variability, which is defined by the interquartile range (IQR) of the saccade endpoints, estimated by the bootstrap method (2000 iterations).

For the analysis, all centrifugal saccades from the correct trials were collected and grouped into 4 types by saccade direction (saccade to intact/intact-to-be or affected/affected-to-be visual field) and period (pre-lesion or post-lesion). We tested whether the saccadic reaction times (SRTs) and saccade endpoints were significantly different, especially when the monkeys made saccades to the target in their affected visual field (left visual field for monkey C, right visual field for monkey T, after lesioning). Both monkeys exhibited longer SRTs after lesioning regardless of the direction of saccades compared to those before lesioning (significant main effect of lesioning by 2-way analysis of variance on rank; monkey C: $F(1, 20320) = 1904.99$, $p < 10^{-10}$; monkey T: $F(1, 19724) = 4726.53$, $p < 10^{-10}$, **a**). The interaction effect was significant in monkey C ($F(1, 20320) = 264.99$, $p < 10^{-10}$), but not in monkey T ($F(1, 19724) = 3.51$, $p = 0.06$). Saccades to the affected field had significantly shorter SRTs in monkey C, but longer SRTs in monkey T, compared to saccades directed toward the intact visual field ($p < 10^{-10}$, *post-hoc* Mann-Whitney test with Bonferroni's correction). Both monkeys showed significant effect of direction of saccade, lesioning and interaction effects on saccade endpoints (2-way analysis of variance on rank; monkey C: $F(1, 20320) = 134.28$ (direction of saccade), 323.7 (lesioning), 2872.52 (interaction), $p < 10^{-10}$ for all; monkey T: $F(1, 19724) = 10.16$ (direction of saccade), 219.17 (lesioning), 24.03 (interaction), $p < 0.005$ for all; **b**), but there was no consistent tendency for saccade to the affected field among two monkeys, other than the endpoint variability (**c**). Asterisk (*) indicates significant difference ($p < 10^{-10}$).

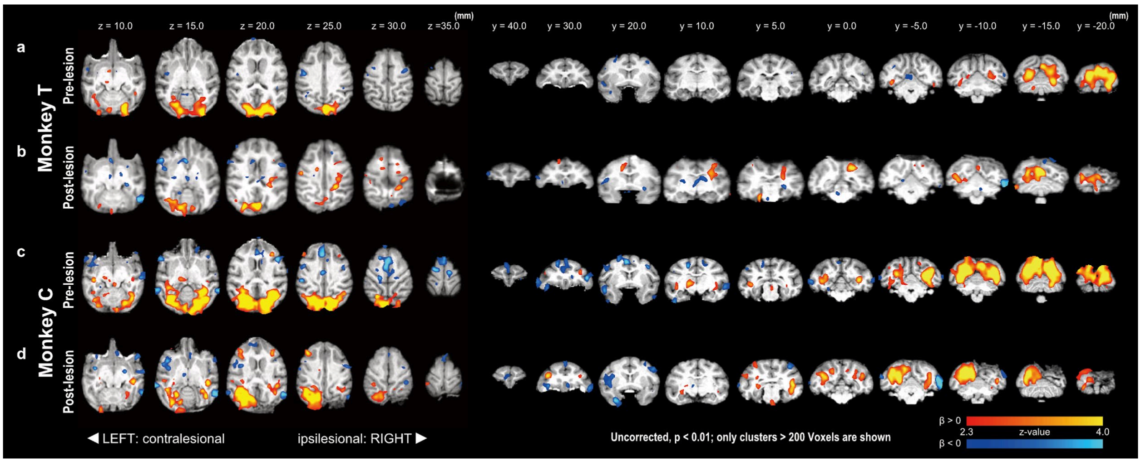

Supplementary Figure 3. Whole brain PET analysis: individual subjects.

Brain areas with a significant relationship to task condition analyzed separately for individual monkeys for each period (pre- and post-lesion). The left and right sides of the brain were flipped to match the side of the lesion (the lesioned side is presented on the right side in this figure). Red-yellow and blue-light blue show a significant positive and negative relationship to task condition, respectively. Only clusters larger than 200 voxels are shown (uncorrected $p < 0.01$). **a** and **b** show the results of monkey T in the pre- and post-lesion periods, respectively. **c** and **d** show the results of monkey C in the pre- and post-lesion periods, respectively.

We also analyzed individual monkeys separately to study further the brain areas that are related to task condition. The data were analyzed in the same manner described previously, except that the 4D data were aligned separately for each monkey using rigid body transformation.

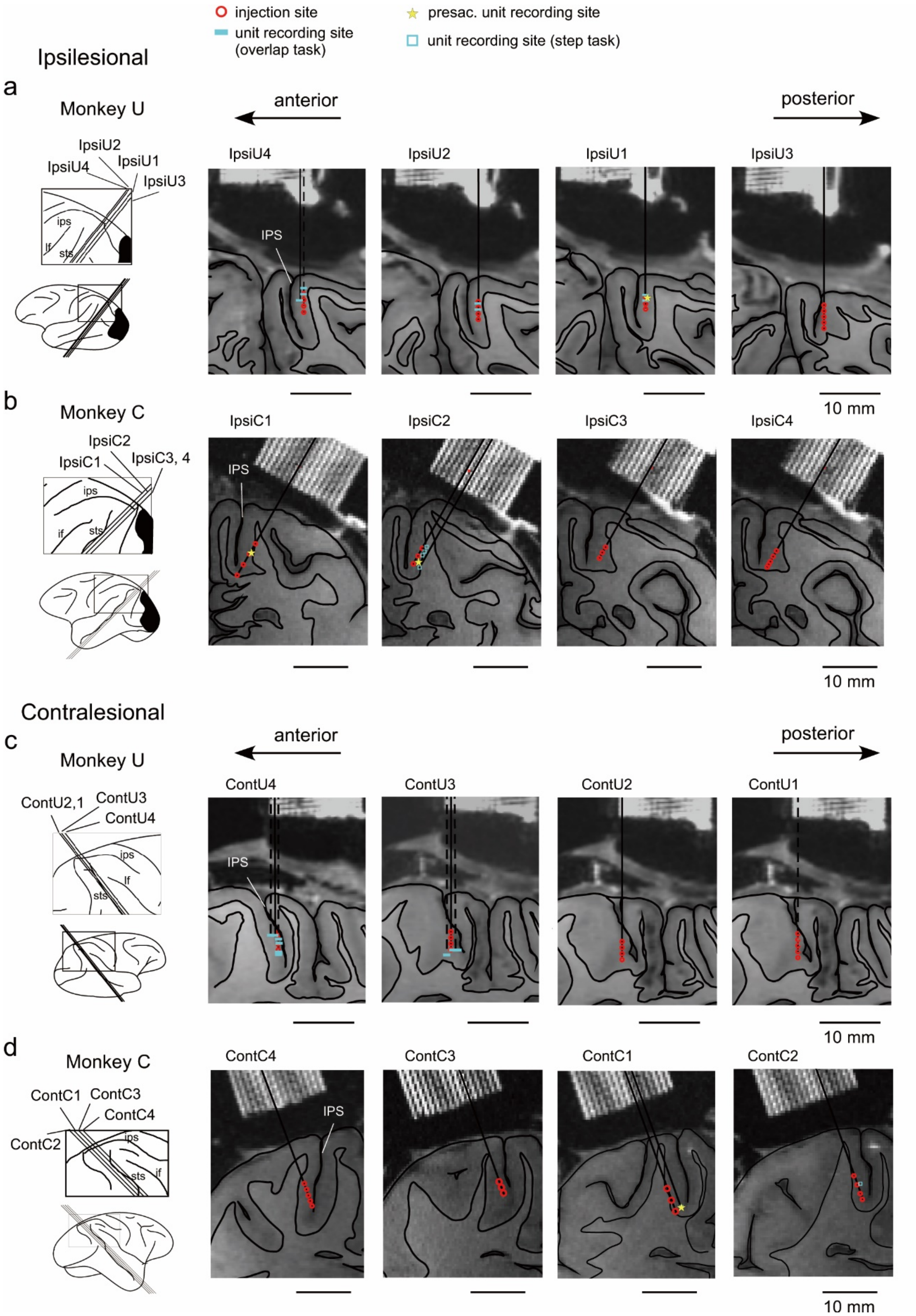

Supplementary Figure 4. Reconstruction of the recording and injection sites in the

lbIPS.

The recording and injection sites in the lbIPS are plotted on the MR images and their traces of monkey U [**a** and **c**] and C [**b** and **d**]. Ipsi- (**a** and **b**) and contralesional (**c** and **d**) sides. The lines in the left panel indicate the level of individual sections shown below with corresponding numbers, projected on the lateral view of each hemisphere. In each section, the sites of recordings or injections in individual experiments are reconstructed on the tracks along the grid according to the depth of tip of the electrode or microsyringe relative to the grid. The tracks along the grid on the MR images are indicated as solid lines (through the grid on the image shown) or dotted lines (through a different coordinate of the grid which shift the insertion holes 0.5mm). The injection sites are indicated with red circles. The recording sites of individual neurons during overlap saccade task and step saccade task are indicated as blue rectangles and blue squares, respectively. The recording sites of neurons with presaccadic activity (Supplementary Figure 6) are indicated as yellow stars. Session numbers (such as ipsiU4 indicated above the individual panels) correspond to those in Supplementary Table 1. All the scale bars indicate 10 mm.

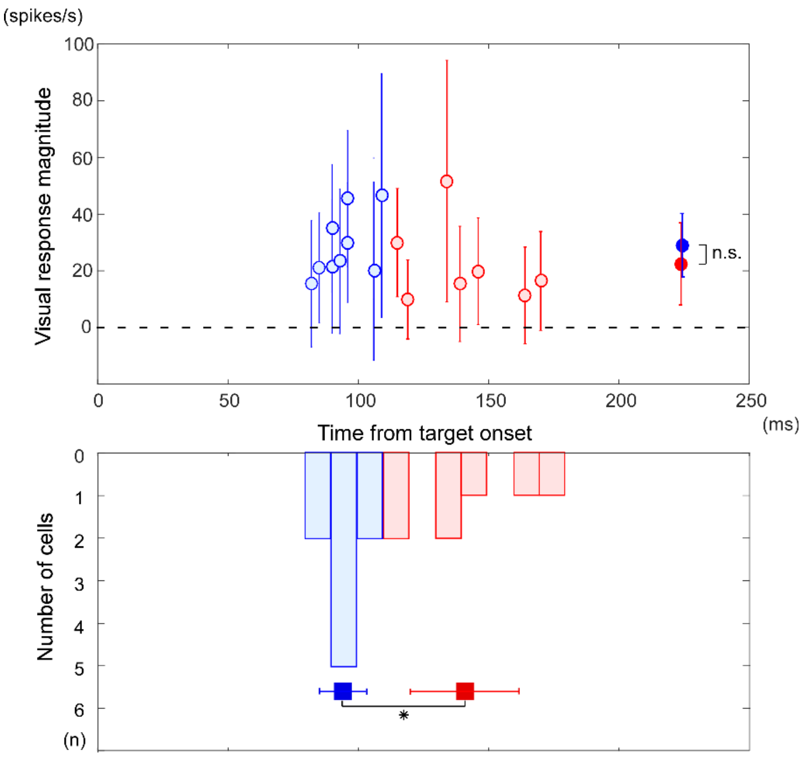

Supplementary Figure 5. Distribution of the latencies and magnitudes of the visual responses in individual lbIPS neurons.

The lower panel shows histograms of visual response latency of ipsi- (red) and contralesional (blue) lbIPS neurons. Open circles in the upper panel indicate the visual response magnitude (mean ± SD) of individual neurons in the ipsi- (red) and contralesional (blue) lbIPS. The mean of each group of lbIPS neurons is indicated as a filled box for the latency ($p < 0.05$ by Welch's non-paired t-test. ipsilesional lbIPS: $n = 7$, contralesional lbIPS: $n = 9$. Error bars indicate SD) and a filled circle for the visual response magnitude ($p > 0.05$ by non-paired t-test. ipsilesional lbIPS: $n = 7$, contralesional lbIPS: $n = 9$. Error bars indicate SD) with the corresponding color.

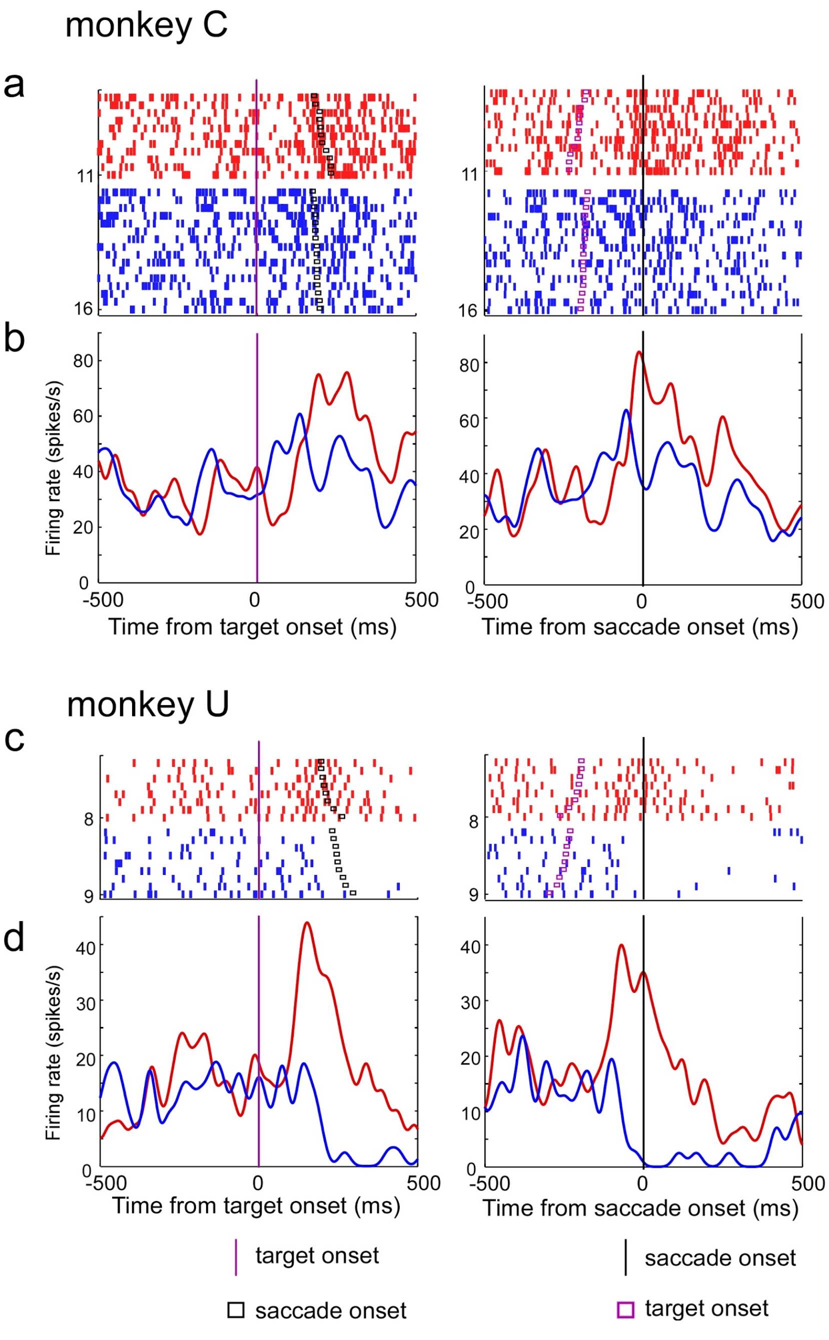

Supplementary Figure 6. Presaccadic activity of ipsilesional lbIPS neurons.

Two examples of ipsilesional lbIPS neurons recorded in monkey C (**a** and **b**) and monkey U (**c** and **d**), which showed presaccadic activity in the step saccade task. **a** and **c** show the spike rastergram. **b** and **d** show the averaged spike density functions. In case of the left panels, the data are aligned to the target onset. In case of the right panels, the data are

aligned to the saccade onset. Red dots and lines indicate the neural activity when the target was presented in their response field (affected visual field), while blue dots and lines indicate the neural activity when the target was presented outside the response field (intact visual field). In case of the neuron in **a** and **b**, the peak of phasic activity starting immediately before the saccade onset was sharper and higher when the data are aligned to the saccade onset. In case of the neuron in **c** and **d**, the second peak emerged when the data are aligned in the saccade onset and started before the saccade onset. Therefore, both neurons could be considered to exhibit the presaccadic activity.

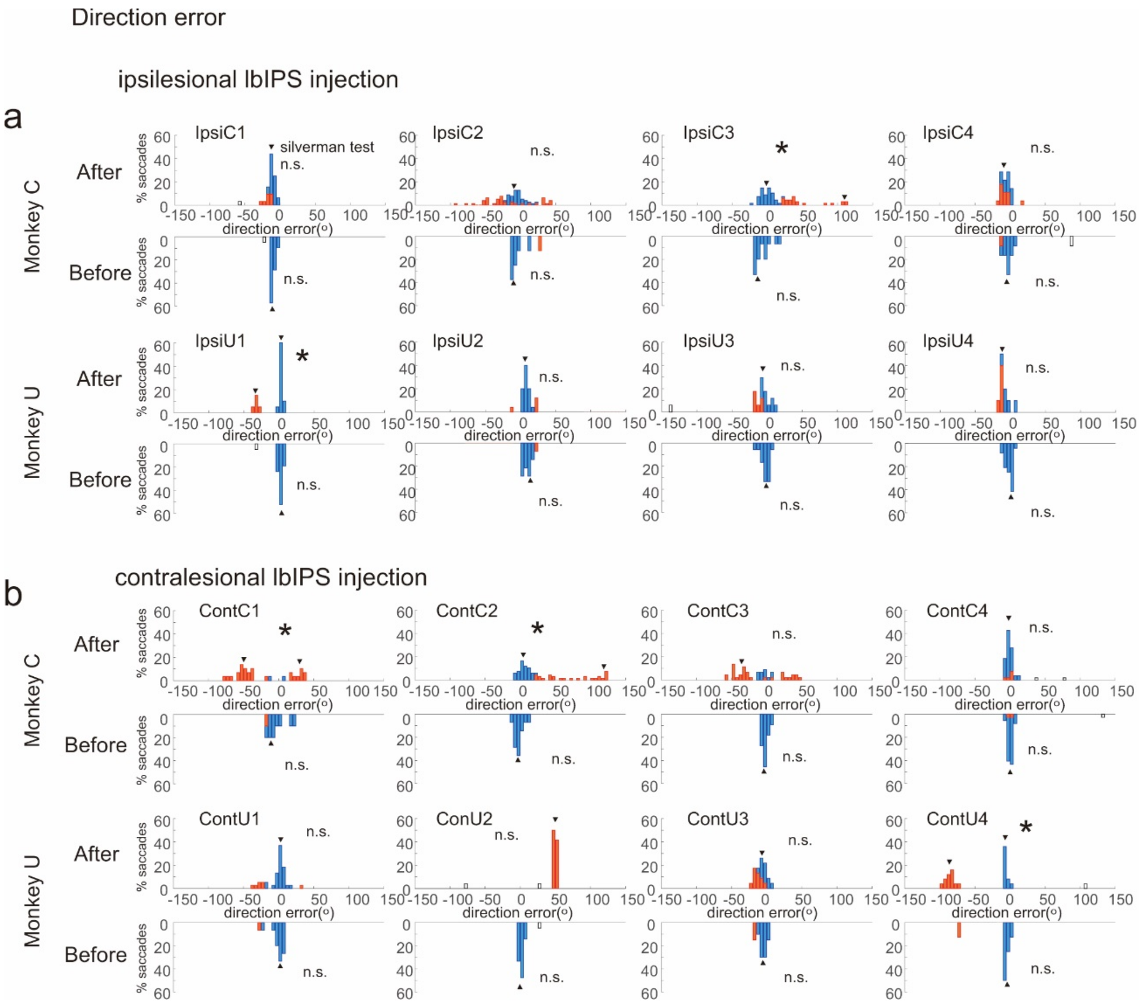

Supplementary Figure 7. Distributions of direction error of saccade endpoints.

a, b. Distributions of endpoint direction relative to the target, before and after the muscimol injection into the ipsi- (**a**) or contralesional lbIPS (**b**) in individual sessions are plotted. Distributions without an outlier (indicated by the open columns, see Methods) were tested by Silverman's test. An asterisk indicates the significance for multimodality by Silverman's test ($p < 0.05$). Triangles mark the position of the local mode along the horizontal axis estimated by Silverman's test. Blue and red columns indicate the correct and error saccades, respectively. Open columns indicate the saccades removed from the analysis as outliers (see Methods).

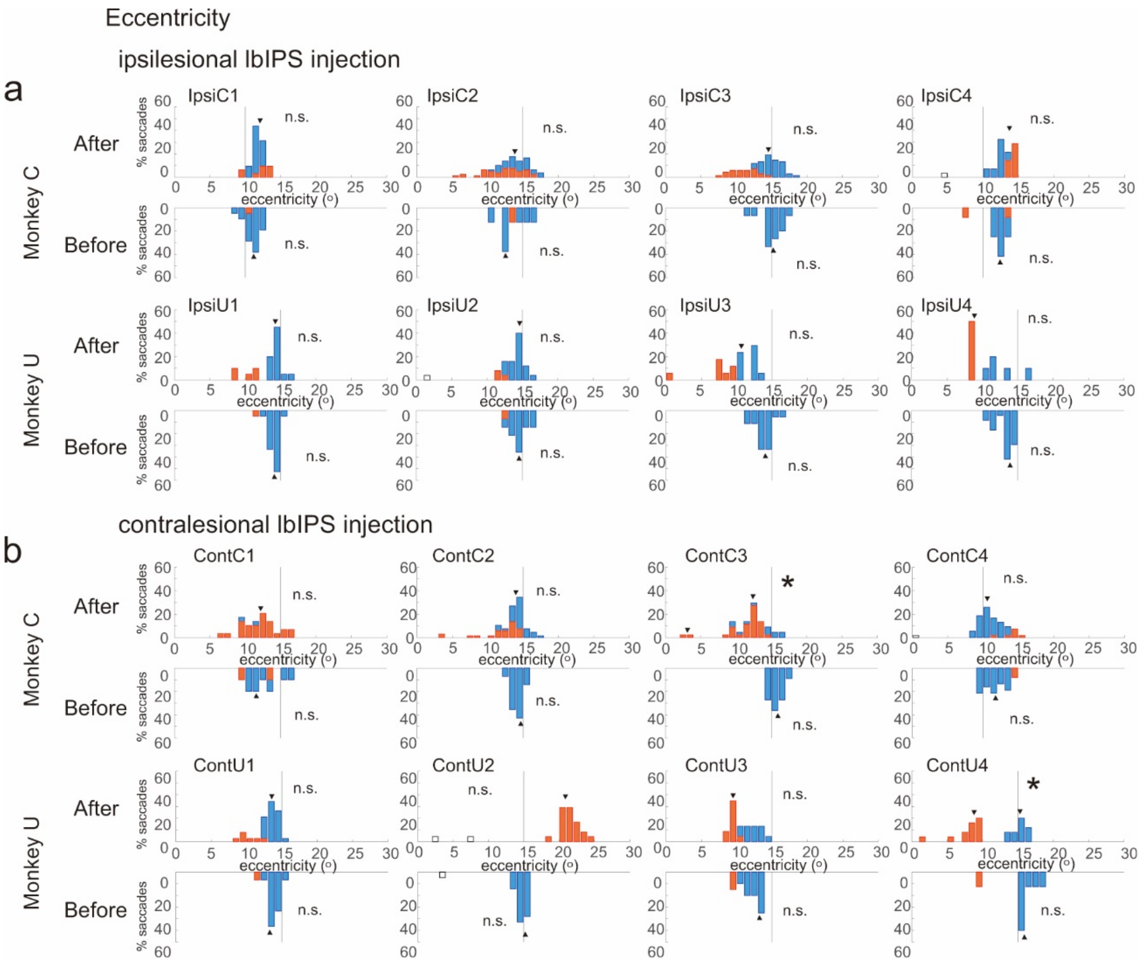

Supplementary Figure 8. Distributions of eccentricity of saccadic endpoints.

a, b. Distributions of endpoint eccentricity before and after muscimol injection into the ipsi- (**a**) or contralesional lbIPS (**b**) in individual sessions are plotted. In each panel, a solid line indicates the eccentricity of the target from the fixation point. The same arrangement as Supplementary Figure 7.

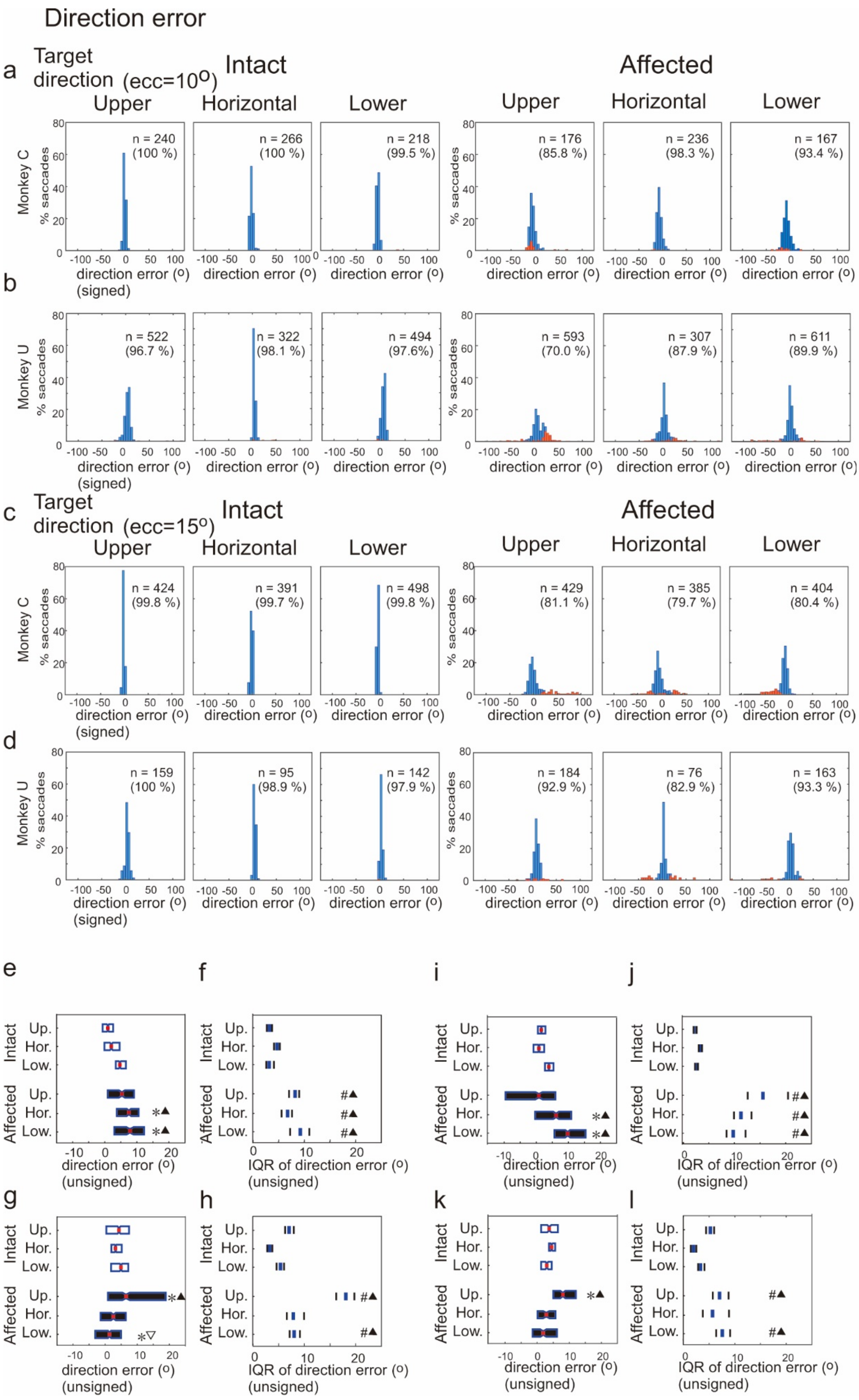
Direction error
a
Target direction (ecc=10°)
Intact
Affected
Upper
Horizontal
Lower
Monkey C
% saccades
n = 240 (100 %)
n = 266 (100 %)
n = 218 (99.5 %)
n = 176 (85.8 %)
n = 236 (98.3 %)
n = 167 (93.4 %)
direction error (°) (signed)
direction error (°)
b
Monkey U
n = 522 (96.7 %)
n = 322 (98.1 %)
n = 494 (97.6%)
n = 593 (70.0 %)
n = 307 (87.9 %)
n = 611 (89.9 %)
c
Target direction (ecc=15°)
n = 424 (99.8 %)
n = 391 (99.7 %)
n = 498 (99.8 %)
n = 429 (81.1 %)
n = 385 (79.7 %)
n = 404 (80.4 %)
d
n = 159 (100 %)
n = 95 (98.9 %)
n = 142 (97.9 %)
n = 184 (92.9 %)
n = 76 (82.9 %)
n = 163 (93.3 %)
e
f
i
j
g
h
k
l
Intact
Affected
Up.
Hor.
Low.
direction error (°) (unsigned)
IQR of direction error (°) (unsigned)

Supplementary Figure 9. Effects of V1 lesion on the accuracy of saccades; analysis of datasets during the control session before the lbIPS inactivation experiments.

Directions of saccade endpoints relative to the target (direction error) were compared between saccades towards the intact and affected visual fields. Because the data of lbIPS inactivation was recorded long after the V1 lesion and in the different experimental settings, the direct comparison of saccade accuracy on the same side of the visual field between pre and post V1 lesion was not possible. Therefore, to estimate how the V1 lesion by itself affected the accuracy of saccades, we compared the direction errors in the intact visual field and affected visual field.

a, b, c and **d**. Distributions of direction errors (signed) in the intact visual field (left three panels) and the affected visual field (right three panels) in the monkey C (**a**, **c**) and monkey U (**b**, **d**). For direction error (signed), upper direction error was indicated as plus and lower as minus, respectively. Blue and red columns indicate the correct and error saccades, respectively (see Methods). These saccade endpoints were collected during the step saccade task. The target locations were set at various points in the visual field, to match with the most effective site in the later lbIPS inactivation experiments. Each panel includes the data of saccade groups with the target directions as follows. Intact, upper: 25° to 65°, horizontal: 340° to 20°, lower: 295° to 335°. Affected, upper: 115° to 155°, horizontal: 160° to 200°, lower: 205° to 245°. The number of trials and the success rate are presented in the insets of individual panels.

e, g, i and **k.** Median of direction errors (unsigned, red line) and the IQR of each dataset in **f, h, j** and **l**, respectively, were plotted. Direction error (unsigned): to compare how much saccades were deviated from the target locations, the data points were flipped relative to zero if the median of the error was negative value. Here, the labels along the vertical axis indicate the target direction. Open boxes indicate data with the targets in the intact field. Black filled boxes indicate data with the targets in the affected field. Differences between each distribution of direction error were tested by the Steel-Dwass test (by R package: NSM3 or "http://aoki2.si.gunma-u.ac.jp/R/src/Steel-Dwass.R", encoding="euc-jp"). In this test, the dataset of each target direction in the affected visual field was compared with other 5 datasets (3 target directions in the intact visual field and 2 other target directions in the affected visual field), and for the comparison of intact vs

affected visual field, we focused on the comparison with 3 target directions in the intact field. We conducted the test on 3 target groups for each 2 eccentricities (10 and 15°) in 2 animals, that is, totally 12 target groups. It was found that direction errors of saccades towards 6/12 target groups in the affected visual field were larger than all of three target groups in the intact visual field with same eccentricity (*▲: Steel-Dwass test, $p < 0.05$). In one case with *▽the direction error in the affected visual field was smaller than those on the intact side. **f, h, j** and **l.** Distribution of saccade direction error in the affected visual field was compared with those in the intact visual field also using the IQR and its 95% confidence intervals. IQR (blue dot) and the 95% confidence interval (black line) of each dataset in **a, b, c** and **d**, are plotted. #▲ placed right to the IQR on the affected side indicated that the IQR was significantly larger than all upper edges of 95% confidence interval of three target groups on the intact side (10/12). All these results fit our previous study which showed saccades became less accurate after V1 lesion (Yoshida et al, 2008) and our current datasets obtained in the round saccade task (see Supplementary Figure 2). These datasets of control sessions were not collected in the same experimental protocol as the datasets of lbIPS inactivation experiments. Therefore, it was not possible to directly compare this result with the effects of the lbIPS inactivation (Supplementary Figure 7). However, considering the large reductions of success rate and the increased variance of the saccade endpoint distribution (the multimodal distribution and increased IQR) during the lbIPS inactivation (Figs.6 and 7, Supplementary Figure 7, Supplementary Table1, 2 and 3), it was clear that the performance of visually guided saccade task were impaired by the lbIPS inactivation.

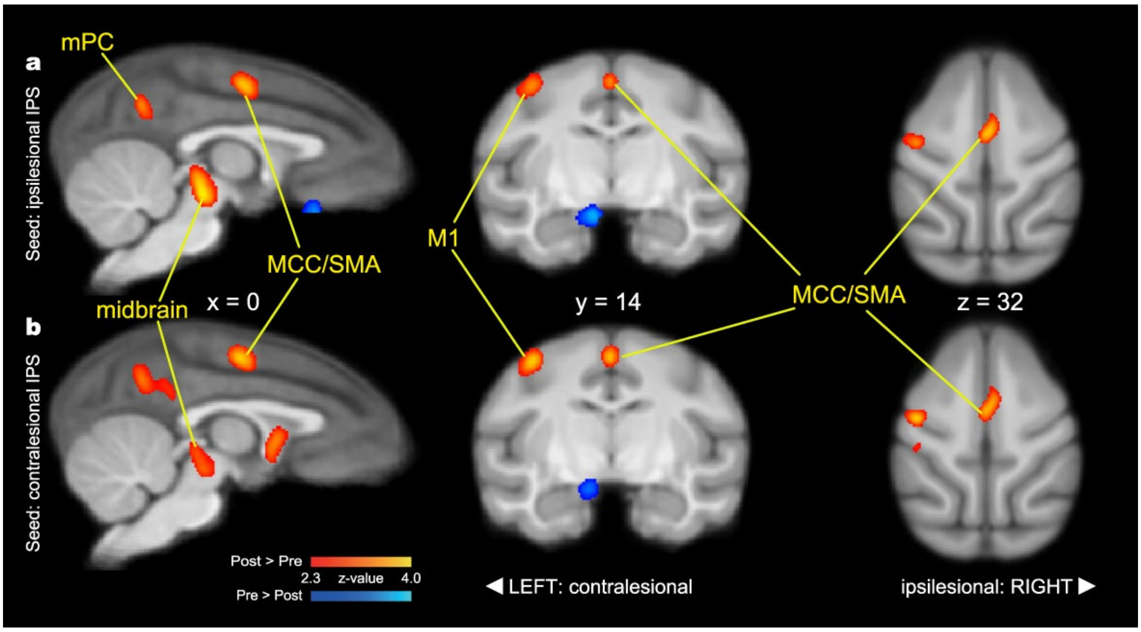

Supplementary Figure 10. The effective functional connectivity of IPS associated with task and lesion.

The figure shows three orthogonal sections of brain (gray color) and areas with significant change in psychophysiological interactions (PPI) with ipsilesional IPS (**a**) and contralesional IPS (**b**) in post-lesion period as compared with pre-lesion period. Midcingulate cortex (MCC)/Supplementary motor area (SMA), midbrain, medial parietal cortex, and M1 showed significant PPI with both ipsi- and contralesional IPS areas, specifically in the post-lesion period.

The PPI searched the areas whose rCBF values are correlated with that of a seed region (e.g., ipsi- or contralesional IPS) and associated by the other factors (e.g., task and lesion). Seed regions of ipsi- and contralesional IPS were defined by the 5x5x5 voxels around local maxima of Post > Pre contrast in task relationship (as shown in Fig.4c- d). The first-level PPIs were analyzed using FEAT, separately for each subject, period, and seed region. Regional CBFs were regressed by variables of task condition (1, 2, 3, 4, or 6) and rCBF values in seed region (ipsi- or contralesional IPS), and an interaction between task condition and seed rCBF. We also used a regressor for global signal to remove its effect. Then the contrasts of the PPI were compared between the pre- and post-lesion periods in both monkeys to search areas with higher-level fixed effects of PPI. The statistical

threshold was set at $Z > 2.3$, and each cluster of voxels with a higher Z-value was tested using Gaussian random field theory ($p < 0.05$). Details are shown in Supplementary Table 4.

monkey C

ipsilesional lbIPS Session	Num. of injections * Conc. (µg/µl)	Target location: direction (°) affected direction	direction (°) intact direction	ecc (°)	Success rates (%), (Number of trials): affected before	affected after	intact before	intact after	SRT (ms), (IQR): affected before	affected after	affected difference [before - after]	intact before	intact after	intact difference [before - after]
IpsiC1	4 * 2	255	75	10	95 (21)	72 (32)	100 (21)	100 (38)	191 (6)	198 (16)	*	185 (11)	175 (10)	*
IpsiC2	3 * 5	195	60	15	87 (8)	51(79)	100 (10)	100 (60)	202 (27)	210 (28)	n.s.	178 (10)	176 (24)	n.s.
IpsiC3	3 * 5	130	85	15	100 (15)	65 (68)	100 (9)	100 (83)	194 (17)	210 (29)	*	182 (10)	191 (13)	n.s.
IpsiC4	5 * 5	125	350	10	83 (12)	54 (28)	100 (9)	100 (28)	196 (12)	196 (12)	n.s.	177 (9)	179 (9)	n.s.

contralesional lbIPS Session	Num. of injections * Conc. (µg/µl)	Target location: direction (°) affected direction	direction (°) intact direction	ecc (°)	Success rates (%), (Number of trials): affected before	affected after	intact before	intact after	SRT (ms), (IQR): affected before	affected after	affected difference [before - after]	intact before	intact after	intact difference [before - after]
ContC1	3 * 2	200	65	15	80 (10)	7 (29)	100 (8)	100 (29)	210 (28)	223 (36)	n.s.	188 (7)	178 (10)	*
ContC2	4 * 5	120	30	15	100 (14)	60 (67)	100 (17)	100 (77)	210 (27)	210 (19)	n.s.	171 (20)	184 (20)	n.s.
ContC3	3 * 5	195	15	15	100 (11)	28 (44)	100 (9)	96 (28)	213 (25)	215 (32)	n.s.	163 (20)	183 (35)	n.s.
ContC4	5 * 2	110	335	10	92 (37)	85 (54)	97 (29)	100 (47)	208 (10)	206 (12)	n.s.	177 (16)	185 (13)	*

monkey U

ipsilesional lbIPS Session	Num. of injections * Conc. (µg/µl)	Target location: direction (°) affected direction	direction (°) intact direction	ecc (°)	Success rates (%), (Number of trials): affected before	affected after	intact before	intact after	SRT (ms), (IQR): affected before	affected after	affected difference [before - after]	intact before	intact after	intact difference [before - after]
IpsiU1	3 * 5	210	345	15	95 (21)	75 (20)	100 (11)	95 (22)	214 (20)	205 (27)	n.s.	222 (22)	220 (35)	n.s.
IpsiU2	4 * 5	120	75	15	93 (14)	84 (25)	100 (15)	100 (27)	207 (11)	204 (23)	n.s.	180 (10)	182 (7)	n.s.
IpsiU3	5 * 5	165	300	15	100 (18)	59 (17)	100 (23)	89 (18)	203 (11)	206 (18)	n.s.	210 (20)	230 (22)	*
IpsiU4	4 * 5	165	300	15	100 (24)	50 (10)	100 (30)	100 (11)	200 (14)	195 (27)	n.s.	216 (19)	231 (12)	*

contralesional lbIPS Session	Num. of injections * Conc. (µg/µl)	Target location: direction (°) affected direction	direction (°) intact direction	ecc (°)	Success rates (%), (Number of trials): affected before	affected after	intact before	intact after	SRT (ms), (IQR): affected before	affected after	affected difference [before - after]	intact before	intact after	intact difference [before - after]
ContU1	5 * 5	195	60	15	93 (15)	82 (38)	100 (34)	100 (27)	218 (15)	218 (13)	n.s.	179 (9)	184 (13)	*
ContU2	4 * 5	255	30	15	95 (21)	0 (24)	100 (26)	100 (22)	193 (11)	209 (27)	*	183 (10)	182 (10)	n.s.
ContU3	4 * 5	165	75	15	85 (20)	52 (23)	100 (14)	100 (23)	196 (16)	199 (18)	n.s.	189 (18)	193 (20)	n.s.
ContU4	4 * 5	240	285	15	88 (8)	48 (25)	100 (8)	90 (31)	217 (23)	213 (39)	n.s.	219 (16)	225 (17)	*

Supplementary Table 1. List of muscimol injection sessions and summary of their results.

SRTs, median saccadic reaction times before and after injection in individual sessions; IQR, interquartile range. The asterisks indicate a significant difference in SRTs between before and after injection ($p < 0.05$ by the Wilcoxon rank sum test). Target location: location with the smallest [Success Rate (After) - Success Rate (Before)] in each session.

monkey C

ipsilesional lbIPS

Session	Target location affected direction (°)	ecc (°)	Direction error (signed) (°) Median (Number of trials) before	after	difference before-after	Direction error (unsigned) (°) difference before-after	IQR before	after
IpsiC1	255	10	-10.7 (21)	-12.0 (32)	n.s.	n.s.	3.2	6.1
IpsiC2	195	15	-7.8 (8)	-9.0 (79)	n.s.	n.s.	18.3	28.5
IpsiC3	130	15	-10.0 (15)	4.3 (62)	*	n.s.	13.9	29.5
IpsiC4	125	10	-4.0 (12)	-6.3 (28)	n.s.	n.s.	11	9.6

contralesional lbIPS

Session	Target location affected direction (°)	ecc (°)	Direction error (signed) (°) Median (Number of trials) before	after	difference before-after	Direction error (unsigned) (°) difference before-after	IQR before	after
ContC1	200	15	-8.0 (10)	29.6 (10)	*	*	18.4	14.8
ContC2	120	15	-3.1 (14)	9.1 (54)	*	*	5.7	22.4
ContC3	195	15	-3.6 (11)	-12.1 (44)	n.s.	n.s.	5.5	43.8
ContC4	110	10	0.3 (37)	-0.9 (54)	n.s. (p=0.08)	n.s.	4.8	5.1

monkey U

ipsilesional lbIPS

Session	Target location affected direction (°)	ecc (°)	Direction error (signed) (°) Median (Number of trials) before	after	difference before-after	Direction error (unsigned) (°) difference before-after	IQR before	after
IpsiU1	210	15	2.9 (21)	2.6 (10)	n.s.	n.s.	5.6	3.2
IpsiU2	120	15	9.9 (14)	7.6 (25)	n.s.	n.s.	10.2	6.6
IpsiU3	165	15	-1.6 (18)	-6.5 (17)	n.s.	n.s.	7.2	13.3
IpsiU4	165	15	-1.4 (24)	-10.9 (10)	*	*	7.4	7.7

contralesional lbIPS

Session	Target location affected direction (°)	ecc (°)	Direction error (signed) (°) Median (Number of trials) before	after	difference before-after	Direction error (unsigned) (°) difference before-after	IQR before	after
ContU1	195	15	2.1 (15)	2.2 (38)	n.s.	n.s.	6.9	7.8
ContU2	255	15	0.9 (21)	49.6 (24)	*	*	4.5	3.3
ContU3	165	15	-5.9 (20)	-6.8 (23)	n.s.	n.s.	6.9	11.1
ContU4	240	15	-6.3 (8)	-5.9 (13)	n.s.	n.s.	6.1	2.7

Supplementary Table 2. Effects of lbIPS inactivation on the on-target saccades: direction.

Comparison of direction errors of on-target saccades before and during the lbIPS inactivation. Direction error (unsigned): to compare how much saccades were deviated from target location, the data points were flipped relative to zero if the median of the error was negative value.

The asterisk (*) indicates significant difference between before and after muscimol injection ($p < 0.05$ by the Wilcoxon rank sum test). “n.s.” indicates “not significant”.

Target location : location with the smallest [Success Rate (After) - Success Rate (Before)] in each session.

monkey C

ipsilesional lbIPS

Session	Target location		Eccentrricity (°)			Eccentricity error (unsigned) (°)	IQR	
	affected direction	ecc (°)	Median (Number of trials) before	after	difference before-after	difference before-after	before	after
IpsiC1	255	10	11.1 (21)	11.8 (32)	*	*	1.8	1.3
IpsiC2	195	15	13.2 (8)	13.4 (79)	n.s.	n.s.	2.5	3.6
IpsiC3	130	15	15.3 (15)	14.1 (68)	*	n.s.	1.9	3.2
IpsiC4	125	10	12.2 (12)	13.0 (28)	n.s. (p=0.07)	n.s. (p=0.07)	1.4	2.1

contralesional lbIPS

Session	Target location		Eccentrricity (°)			Eccentricity error (unsigned) (°)	IQR	
	affected direction	ecc (°)	Median (Number of trials) before	after	difference before-after	difference before-after	before	after
ContC1	200	15	12.1 (10)	12.2 (29)	n.s.	n.s.	2.9	3.6
ContC2	120	15	14.5 (14)	13.8 (67)	n.s.	n.s.	0.9	1.7
ContC3	195	15	15.8 (11)	12.3 (42)	*	*	1.9	2.1
ContC4	110	10	11.6 (37)	11.0 (54)	n.s.	n.s.	3.1	2.5

monkey U

ipsilesional lbIPS

Session	Target location		Eccentrricity (°)			Eccentricity error (unsigned) (°)	IQR	
	affected direction	ecc (°)	Median (Number of trials) before	after	difference before-after	difference before-after	before	after
IpsiU1	210	15	14.0 (21)	14.1 (20)	n.s.	n.s.	0.9	2.2
IpsiU2	120	15	14.4 (14)	14.3 (25)	n.s.	n.s.	1.5	2
IpsiU3	165	15	13.9 (18)	10.6 (17)	*	*	1.4	4.2
IpsiU4	165	15	13.8 (24)	10.0 (10)	*	*	2.2	3.2

contralesional lbIPS

Session	Target location		Eccentrricity (°)			Eccentricity error (unsigned) (°)	IQR	
	affected direction	ecc (°)	Median (Number of trials) before	after	difference before-after	difference before-after	before	after
ContU1	195	15	13.7 (15)	13.3 (38)	n.s.	n.s.	1.3	1.7
ContU2	255	15	14.8 (21)	21.2 (24)	*	*	1.1	1.8
ContU3	165	15	12.3 (20)	10.4 (23)	*	*	2.5	2.8
ContU4	240	15	15.6 (8)	15.5 (12)	n.s.	n.s.	1.6	1.4

Supplementary Table 3. Effects of lbIPS inactivation on the on-target saccades: eccentricity.

Comparison of eccentricity errors of on-target saccades before and during the lbIPS inactivation. Eccentricity error (unsigned): to compare how much saccades were deviated from target location, the data points were flipped relative to zero if the median of the error was negative value.

Asterisk (*) indicates significant difference between before and after muscimol injection ($p < 0.05$ by the Wilcoxon rank sum test). “n.s.” indicates “not significant”.

Target location: location with the smallest [Success Rate (After) - Success Rate (Before)] in each session.

Distribution of saccade endpoints during the lbIPS inactivation was sometimes unimodal and sometimes multimodal. Multimodality of the distributions after the inactivation was

statistically determined in Fig.7 and Supplementary Figures 7 and 8 using the Silverman's test. Here, we focused on the on-target saccades. The saccades in the mode judged as being remote from the target location by the Silverman's test in multimodal case were considered as "off-target" saccades and the remaining saccades were judged as "on-target". If the distribution was unimodal, all the saccades were regarded as on-target. Supplementary Table 2 and 3 show the results comparing the on-target saccade endpoint errors before and after the muscimol injection (Supplementary Table 2: direction error, Supplementary Table 3: eccentricity error), focusing on on-target saccades. Here, even after excluding those off-target saccades, the direction and the eccentricity of saccades became less accurate after the inactivation in 4 out of 16 experiments and 6 out of 16 experiments, respectively (indicated by * in Supplementary Table 2 and Supplementary Table 3). The effects were observed in 4 experiments in monkey C (direction error: contC1 and contC2: eccentricity error: ipsiC1 and contC3), and 4 experiments in monkey U (both errors: ipsiU4 and contU2; eccentricity error: ipsi U3 and contU3).

Pre-Post Comparisons
seed ROI: Ipsilesional IPS
Post > Pre

Cluster Index	Voxels	Z-max	COG (x, y, z) (mm)	Region	Local maxima (x, y, z) (mm)	Z-value	Locus
4	1311	4.52	1.0, 6.4, 10.5	midbrain	1.5, 6, 10	4.52	contralesional midbrain
3	864	3.67	-0.8, 16.7, 32.7	MCC/SMA	-0.5, 16, 32.5	3.67	MCC
2	649	3.34	1.2, -5.7, 26.7	medial parietal cortex	0.5, -6.5, 28	3.34	contralesional mPC
1	507	3.26	17.6, 14.0, 32.6	contralesional M1	17.0, 13.5, 33.0	3.26	M1

Pre > Post

Cluster Index	Voxels	Z-max	COG (x, y, z) (mm)	Region	Local maxima (x, y, z) (mm)	Z-value	Locus
7	1218	2.84	-4.0, -10.1, 22.4	ipsilesional V2/PO	-2.5, -12.0, 25.0	2.84	ipsilesional V2
					-3.5, -7.5, 20.0	2.83	ipsilesional V2
					-5.0, -5.0, 15.5	2.63	ipsilesional V2
					-5.5, -14.0, 24.5	2.61	ipsilesional V2
					-5.0, -10.5, 28.5	2.55	ispilesional PO
6	669	3.55	5.1, 14.2, 4.1	contralesional OT	4.0, 13.5, 4.5	3.55	contralesional OT
5	447	3.27	18.1, 26.0, 17.1	cotralesional precentral gyrus	18.0, 26.0, 17.0	3.27	cotralesional precentral gyrus
4	423	2.96	7.0, -19.9, 17.6	contralesional V2	6.5, -20, 17.5	2.96	contralesional V2
3	416	2.93	10.4, -3.4, 22.2	contralesional MT	10.0, -3.5, 22.5	2.93	contralesional MT
2	331	3.03	11.0, -7.0, 7.0	contralesional CB	10.5, -6.0, 7.0	3.03	contralesional CB
					13.0, -9.5, 6.0	2.68	contralesional CB
1	309	3.52	-1.0, 30.4, 5.9	OMPFC	-1.0, 31.0, 5.0	3.52	OMPFC

seed ROI: contralesional IPS
Post > Pre

Cluster Index	Voxels	Z-max	COG (x, y, z) (mm)	Region	Local maxima (x, y, z) (mm)	Z-value	Locus
9	1050	3.67	-0.9, 16.4, 33.1	MCC/SMA	0, 15, 33	3.67	MCC
8	964	3.11	0.9, -4.8, 26.9	medial parietal cortex	0.5, -6.5, 28	3.11	contralesional mPC
					1.0, -1.5, 26.0	2.67	contralesional mPC
7	773	3.83	1.2, 6.2, 10.7	midbrain	1.5, 6.0, 10.5	3.83	contralesional midbrain
6	633	3.79	17.4, 13.7, 32.5	contralesional M1	17, 13.5, 32.5	3.79	contralesional M1
5	577	3.32	-0.6, 22.6 14.6	septal area/anterior commissure	-1.0, 23.0, 15.5	3.32	ipsilesional septral area
4	457	3.2	-8.3, 16.7, -5.4	ipsilesional entorhinal cortex	-8.5, 17.0, -5.0	3.2	ipsilesional entorhinal cortex
3	447	3.28	-24.5, -1.3, 27.2	ipsilesional area TPO	-24.5, -1, 26.5	3.28	ipsilesional area TPO
2	389	3.24	-4.0, 43.9, 19.2	ipsilesional preforntal cortex	-3.5, 44, 19.5	3.24	ipsilesional preforntal cortex
1	359	3	18.1, 5.2, 29.4	ipsilesional anterior IPS	18.5, 4.5, 28.5	3	contralesional infreior parietal lobule
					17, 6.5, 32	2.5	contralesional somatosensory cortex

Pre > Post

Cluster Index	Voxels	Z-max	COG (x, y, z) (mm)	Region	Local maxima (x, y, z) (mm)	Z-value	Locus
7	1092	3.43	-3.9, -7.0, 18.9	ipsilesional V2	-3.5, -7.5, 19.5	3.43	ipsilesional V2
6	1068	3.28	6.7, -1.9, 20.8	contralesional VIP/PCC	9.0, -3.5, 23.0	3.28	contralesional VIP
					3.5, -0.5, 17.5	3.07	contralesional PCC
					6.0, -0.5, 20.5	2.79	
5	585	3.36	18.9, 26.3, 17.1	contralesional precentral gyrus	19.0, 26.5, 17.0	3.36	contralesional precentral gyrus
4	544	3.21	7.2, -19.1, 16.3	contralesional V2	7.0, -19.0, 15.5	3.21	contralesional V2
3	511	3.43	10.9, -5.8, 7.4	contralesional CB	10.5, -5.5, 7.0	3.43	contralesional CB
					13.0, -9.5, 6.0	2.52	contralesional CB
2	443	3.18	5.1, 13.7, 4.6	contralesional OT	4.5, 13.0, 5.0	3.18	contralesional OT
1	292	2.92	8.4, 29.9, 28.3	contralesional PMd	8.5, 29.5, 28.5	2.92	contralesional PMd

Supplementary Table 4. The effective functional connectivity of IPS associated with task and lesion. Clusters with significant change in PPI in the post-lesion period compared to the pre-lesion period are shown (left: seed in ipsilesional IPS, right: seed in contralesional IPS). On the x-axis, the lesioned side is indicated with a minus sign.

abbreviations: CB, Cerebellum; IPS, intraparietal sulcus area; M1, primary motor cortex; MCC, midcingulate cortex; mPC, medial parietal cortex; MT, middle temporal area; OMPFC, orbitomedial prefrontal cortex; OT, Optic tract; PCC, posterior cingulate cortex; PMd, dorsal premotor cortex; PO, parieto-occipital area; SMA, supplemetary motor area; TPO, temporo-parieto-occipital region; VIP, ventral intraparietal area.

Supplementary reference

1. Yoshida, M., Takaura, K., Kato, R., Ikeda, T. & Isa, T. Striate Cortical Lesions

Affect Deliberate Decision and Control of Saccade: Implication for Blindsight. *J Neurosci* **28**, 10517–10530 (2008).
