# Supplementary material for: The posterior parietal cortex contributes to visuomotor processing for saccades in blindsight macaques
Source: Commun Biol. 2021 Mar 4;4:278. doi: 10.1038/s42003-021-01804-z (PMC7933420; doi:10.1038/s42003-021-01804-z)
Supplement: Supplementary file 3 — Description of Supplementary Files [file 42003_2021_1804_MOESM3_ESM.pdf]

Description of Additional Supplementary Files

File name: Supplementary Data 1

Description: An excel file containing the data of figures.

File name: Supplementary Data 2

Description: An excel file containing the data of tables.
