# Supplementary material for: The posterior parietal cortex contributes to visuomotor processing for saccades in blindsight macaques
Source: Commun Biol. 2021 Mar 4;4:278. doi: 10.1038/s42003-021-01804-z (PMC7933420; doi:10.1038/s42003-021-01804-z)
Supplement: Supplementary file 6 — Reporting Summary [file 42003_2021_1804_MOESM6_ESM.pdf]

nature research

Corresponding author(s): Tadashi Isa, Takuro Ikeda

Last updated by author(s): Jan 9, 2021

Reporting Summary

Nature Research wishes to improve the reproducibility of the work that we publish. This form provides structure for consistency and transparency in reporting. For further information on Nature Research policies, see our Editorial Policies and the Editorial Policy Checklist.

Statistics

For all statistical analyses, confirm that the following items are present in the figure legend, table legend, main text, or Methods section.

n/a	Confirmed	
☐	☒	The exact sample size (*n*) for each experimental group/condition, given as a discrete number and unit of measurement
☐	☒	A statement on whether measurements were taken from distinct samples or whether the same sample was measured repeatedly
☐	☒	The statistical test(s) used AND whether they are one- or two-sided<br>*Only common tests should be described solely by name; describe more complex techniques in the Methods section.*
☒	☐	A description of all covariates tested
☒	☐	A description of any assumptions or corrections, such as tests of normality and adjustment for multiple comparisons
☐	☒	A full description of the statistical parameters including central tendency (e.g. means) or other basic estimates (e.g. regression coefficient) AND variation (e.g. standard deviation) or associated estimates of uncertainty (e.g. confidence intervals)
☐	☒	For null hypothesis testing, the test statistic (e.g. F, t, r) with confidence intervals, effect sizes, degrees of freedom and P value noted<br>*Give P values as exact values whenever suitable.*
☒	☐	For Bayesian analysis, information on the choice of priors and Markov chain Monte Carlo settings
☒	☐	For hierarchical and complex designs, identification of the appropriate level for tests and full reporting of outcomes
☒	☐	Estimates of effect sizes (e.g. Cohen's *d*, Pearson's *r*), indicating how they were calculated

Our web collection on statistics for biologists contains articles on many of the points above.

Software and code

Policy information about availability of computer code

Data collection	Behavioral data was collected by TEMPO for Windows (Reflective Computing). Neuronal spiking data were collected by Alpha-Omega Data Acquisition System.
Data analysis	Spatial preprocessing and statistical analysis of the data obtained by PET imaging were performed by using FSL 5.0.9 (FMRIB Software Library, Oxford University) and FEAT.<br>Silverman's test for multimodality was performed using R package (Florian Schwaiger, Hajo Holzmann: https://www.mathematik.uni-marburg.de/~stochastik/R_packages/silvermantest_manual.pdf) (R version 3.6.0).<br>Steel-Dwass test was performed using by R package: NSM3 or "http://aoki2.si.gunma-u.ac.jp/R/src/Steel-Dwass.R", encoding="euc-jp"<br>All other analyses were performed by using MATLAB (Mathworks Inc., USA).

For manuscripts utilizing custom algorithms or software that are central to the research but not yet described in published literature, software must be made available to editors and reviewers. We strongly encourage code deposition in a community repository (e.g. GitHub). See the Nature Research guidelines for submitting code & software for further information.

Data

Policy information about availability of data

All manuscripts must include a data availability statement. This statement should provide the following information, where applicable:

- Accession codes, unique identifiers, or web links for publicly available datasets
- A list of figures that have associated raw data
- A description of any restrictions on data availability

The data that support the findings of this study are available from the corresponding author upon reasonable request.

Field-specific reporting

Please select the one below that is the best fit for your research. If you are not sure, read the appropriate sections before making your selection.

☒ Life sciences ☐ Behavioural & social sciences ☐ Ecological, evolutionary & environmental sciences

For a reference copy of the document with all sections, see nature.com/documents/nr-reporting-summary-flat.pdf

Life sciences study design

All studies must disclose on these points even when the disclosure is negative.

Sample size	Three macaque monkeys were used in this study to confirm the across-subject consistency of he results.
Data exclusions	In PET imaging experiments, those sessions in which subjects showed poor performance and the global count was significantly high or low were discarded, as explained in the method. Neural activity data without sufficient trials for analysis were excluded.
Replication	We tested 2 monkeys for each PET imaging and lbIPS inactivation experiments to study common characteristics in both monkeys
Randomization	Macaque monkeys are costly and difficult for randomized design. Therefore, we did not used randomized deign in this experiment. Instead, we focused on the change between pre-lesion and post-lesion/ intact visual field and affected visual field/ before inactivation and during inactivation. On he other hand, we randomized task condition to prevent subjects to prediction
Blinding	We did not make a blinded experiment and analysis. Because the differences of the performance of visually guided saccades in the V1 lesion monkeys between the intact visual field and the affected visual field, between pre-lesion and post-lesion conditions, between before-inactivation and during-inactivation conditions, were visibly obvious, it was not possible to blindly examine and analyze the performance.

Reporting for specific materials, systems and methods

We require information from authors about some types of materials, experimental systems and methods used in many studies. Here, indicate whether each material, system or method listed is relevant to your study. If you are not sure if a list item applies to your research, read the appropriate section before selecting a response.

Materials & experimental systems

n/a	Involved in the study	
☒	☐	Antibodies
☒	☐	Eukaryotic cell lines
☒	☐	Palaeontology and archaeology
☐	☒	Animals and other organisms
☒	☐	Human research participants
☒	☐	Clinical data
☒	☐	Dual use research of concern

Methods

n/a	Involved in the study	
☒	☐	ChIP-seq
☒	☐	Flow cytometry
☐	☒	MRI-based neuroimaging

Animals and other organisms

Policy information about studies involving animals; ARRIVE guidelines recommended for reporting animal research

Laboratory animals	Three macaque monkeys (monkey C, Macaca mulatta, 6.5 kg male; monkey T, Macaca mulatta, 8.4 kg male; monkey U, Macaca fuscata, female 5.6 kg) were used in this study. The monkeys were purchased from the National Bioresource Project by MEXT (monkey U) or a domestic breeder (monkeys C and T) in Japan.
Wild animals	n/a
Field-collected samples	n/a
Ethics oversight	All experimental procedures were performed in accordance with the National Institutes of Health Guidelines for the Care and Use of Laboratory Animals and Basic Policies for the Conduct of Animals Experiments in Research Institutions by MEXT, Japan, and approved by the Committee for Animal Experiments at the National Institutes of Natural Sciences, Japan and the Central Research Laboratory in Hamamatsu Photonics, Hamamatsu, Japan, and the Ethics Committee on Animal Care and Use of RIKEN Center for Life Science Technologies, Japan.

Note that full information on the approval of the study protocol must also be provided in the manuscript.

Magnetic resonance imaging

Experimental design

Design type | *Indicate task or resting state; event-related or block design.*

Design specifications | *Specify the number of blocks, trials or experimental units per session and/or subject, and specify the length of each trial or block (if trials are blocked) and interval between trials.*

Behavioral performance measures | *State number and/or type of variables recorded (e.g. correct button press, response time) and what statistics were used to establish that the subjects were performing the task as expected (e.g. mean, range, and/or standard deviation across subjects).*

Acquisition

Imaging type(s) | Structural MRI of the living animals and ex-vivo imaging of the postmortem brains

Field strength | 3T

Sequence & imaging parameters | spacing 0.5*0.5*0.5 , 0.4*0.4*0.4,

Area of acquisition | a whole brain scan

Diffusion MRI ☐ Used ☒ Not used

Preprocessing

Preprocessing software | *Provide detail on software version and revision number and on specific parameters (model/functions, brain extraction, segmentation, smoothing kernel size, etc.).*

Normalization | *If data were normalized/standardized, describe the approach(es): specify linear or non-linear and define image types used for transformation OR indicate that data were not normalized and explain rationale for lack of normalization.*

Normalization template | *Describe the template used for normalization/transformation, specifying subject space or group standardized space (e.g. original Talairach, MNI305, ICBM152) OR indicate that the data were not normalized.*

Noise and artifact removal | *Describe your procedure(s) for artifact and structured noise removal, specifying motion parameters, tissue signals and physiological signals (heart rate, respiration).*

Volume censoring | *Define your software and/or method and criteria for volume censoring, and state the extent of such censoring.*

Statistical modeling & inference

Model type and settings | *Specify type (mass univariate, multivariate, RSA, predictive, etc.) and describe essential details of the model at the first and second levels (e.g. fixed, random or mixed effects; drift or auto-correlation).*

Effect(s) tested | *Define precise effect in terms of the task or stimulus conditions instead of psychological concepts and indicate whether ANOVA or factorial designs were used.*

Specify type of analysis: ☐ Whole brain ☐ ROI-based ☐ Both

Statistic type for inference (See Eklund et al. 2016) | *Specify voxel-wise or cluster-wise and report all relevant parameters for cluster-wise methods.*

Correction | *Describe the type of correction and how it is obtained for multiple comparisons (e.g. FWE, FDR, permutation or Monte Carlo).*

Models & analysis

n/a	Involved in the study
☒	☐ Functional and/or effective connectivity
☒	☐ Graph analysis
☒	☐ Multivariate modeling or predictive analysis
